# Anaemia and zidovudine-containing antiretroviral therapy in paediatric antiretroviral programmes in the IeDEA Paediatric West African Database to evaluate AIDS

**DOI:** 10.7448/IAS.16.1.18024

**Published:** 2013-09-17

**Authors:** Lorna A Renner, Fatoumata Dicko, Fla Kouéta, Karen Malateste, Ramatoulaye D Gueye, Edmond Aka, Tanoh K Eboua, Alain Azondékon, Uduok Okomo, Pety Touré, Didier Ekouévi, Valeriane Leroy

**Affiliations:** 1 Department of Child Health, University of Ghana Medical School, Korle Bu Teaching Hospital, Accra, Ghana; 2 Hôpital Gabriel Touré, Bamako, Mali; 3 CHU Charles de Gaulle, Ouagadougou, Burkina Faso; 4 INSERM U897, Institut de Santé Publique, Epidémiologie et Développement (ISPED), Université Bordeaux Segalen, Bordeaux, France; 5 Hôpital d'Enfants Albert-Royer, Dakar, Sénégal; 6 Centre de Prise en charge, de Recherche et de Formation (CePReF), Abidjan, Côte d'Ivoire; 7 Pediatric Ward, CHU de Yopougon, Abidjan, Côte d'Ivoire; 8 UPEIV, Hôpital d'Instruction des Armées, Cotonou, Benin; 9 Medical Research Council Unit, Fajara, Gambia; 10 MTCT Plus Network, Abidjan, Côte d'Ivoire; 11 IeDEA Regional Center, Programme PACCI, Abidjan, Côte d'Ivoire

**Keywords:** antiretroviral therapy, children, cohort studies, HIV infection, adverse reactions, West Africa

## Abstract

**Introduction:**

There is a risk of anaemia among HIV-infected children on antiretroviral therapy (ART) containing zidovudine (ZDV) recommended in first-line regimens in the WHO guidelines. We estimated the risk of severe anaemia after initiation of a ZDV-containing regimen in HIV-infected children included in the IeDEA West African database.

**Methods:**

Standardized collection of data from HIV-infected children (positive PCR<18 months or positive serology ≥18 months) followed up in HIV programmes was included in the regional IeDEA West Africa collaboration. Ten clinical centres from seven countries contributed (Benin, Burkina Faso, Côte d'Ivoire, Gambia, Ghana, Mali and Senegal) to this collection. Inclusion criteria were age <16 years and starting ART. We explored the data quality of haemoglobin documentation over time and the incidence and predictors of severe anaemia (Hb<7g/dL) per 100 child-years of follow-up over the duration of first-line antiretroviral therapy.

**Results:**

As of December 2009, among the 2933 children included in the collaboration, 45% were girls, median age was five years; median CD4 cell percentage was 13%; median weight-for-age *z*-score was −2.7; and 1772 (60.4%) had a first-line ZDV-containing regimen. At baseline, 70% of the children with a first-line ZDV-containing regimen had a haemoglobin measure available versus 76% in those not on ZDV (*p*≤0.01): the prevalence of severe anaemia was 3.0% (*n*=38) in the ZDV group versus 10.2% (*n*=89) in those without (*p*<0. 01). Over the first-line follow-up, 58.9% of the children had ≥1 measure of haemoglobin available in those exposed to ZDV versus 60.4% of those not (*p*=0.45). Severe anaemia occurred in 92 children with an incidence of 2.47 per 100 child-years of follow-up in those on a ZDV-containing regimen versus 4.25 in those not (*p≤*0.01). Adjusted for age at ART initiation and first-line regimen, a weight-for-age *z*-score ≤−3 was a strong predictor associated with a 5.59 times risk of severe anaemia (*p*<0.01).

**Conclusions:**

Severe anaemia is frequent at baseline and guides the first-line ART prescription, but its incidence seems rare among children on ART. Severe malnutrition at baseline is a strong predictor for development of severe anaemia, and interventions to address this should form an integral component of clinical care.

## Introduction

Anaemia is common among HIV-infected children worldwide including Africa [1–3]. Anaemia may impair physical, socio-emotional, neurophysiological functioning and hence decrease the survival [2, 3]. Zidovudine (ZDV), a drug associated with bone marrow suppression [4], is a nucleoside reverse transcriptase inhibitor in first-line antiretroviral therapy (ART) regimens in the WHO guidelines [4]. This raises the realistic possibility of increased anaemia among the children put on a ZDV-containing regimen. The ARROW study shows that regular monitoring is not very important in the first few years of treatment [5].

Laboratory monitoring of children on ART is important so as to detect any toxicities such as anaemia. Single-centre studies from West Africa have documented anaemia in children on ART. However, to date there are no multi-centre data from this region [6, 7]. The paediatric West African Database on AIDS (pWADA) undertook this study to determine the risk of severe anaemia and its predictors after the initiation of ZDV-containing ART or non-ZDV- (Stavudine or Abacavir) containing ART in HIV-infected children. The pWADA collaboration is part of the International Epidemiologic Databases to Evaluate AIDS (IeDEA) programme in West Africa. The overall purpose of the pWADA research programme is to better document the operational access to HIV care and its long-term outcomes among HIV-exposed and HIV-infected children in the West African sub-region. Since the database records several outcomes, it offers the possibility of examining drug-related toxicities.

## Method

This retrospective study evaluated ART-treated children followed up in ten clinical centres in seven countries in West Africa (Benin, Burkina Faso, Cote d'Ivoire, Gambia, Ghana, Mali and Senegal) that contribute standardized data to the pWADA collaboration. All HIV-infected children under 16 years of age starting ART in these programmes from January 2000 to December 2009 were included. In all children, CD4 cell count, CD4 cell percentage, blood haemoglobin level, and blood platelet, granulocyte and leukocyte counts were measured at baseline, then monitored every six months according to the national guidelines. For infants and children, haemoglobin is measured at week 8 after initiation of AZT-containing regimens, or more frequently if symptoms indicate so according to the WHO guidelines [4].

Data obtained at baseline, that is, taken within three months of starting ART and over time were analyzed according to the first-line treatment. The visit windows were defined as the three-month time period around the visit for haemoglobin measurement. Patients were deemed lost to follow-up if they had not attended clinic more than six months after their last visit. Haemoglobin documentation to determine adherence to monitoring guidelines and the incidence of severe anaemia according to the first-line ART and its predictors were investigated. Anaemia was defined as haemoglobin concentration below 10 g/dL and severe anaemia as Hb concentration of less than 7 g/dL using WHO grading [8]. Independent variables looked at were initial first-line ART regimen, baseline Hb, baseline clinical stage, baseline CD4 cell count and percentage, patient age and sex. Continuous variables were compared using the Wilcoxon rank-sum test, and comparisons between two categorical variables were performed using Fisher's exact test. The cumulative probability of severe anaemia was estimated using Kaplan–Meier product limit formulae, with 95% confidence interval (CI). The log-rank test was used for comparisons between groups. The predictive factors associated with severe anaemia were determined using univariate, full model and multi-variate Cox model. The multi-variable analysis was performed by the backward selection procedure. This procedure begins with a model including all variables associated with severe anaemia with a *p*-value below 0.20 in the univariate Cox model analysis. If any predictors are above a 5% *p*-value, the predictor with the highest *p*-value is removed from the model. This process continues until the remaining variables meet the criterion.

The participating centres from their various institutional review boards obtained ethical clearance for the collaboration.

## Results

**Figure 1:**
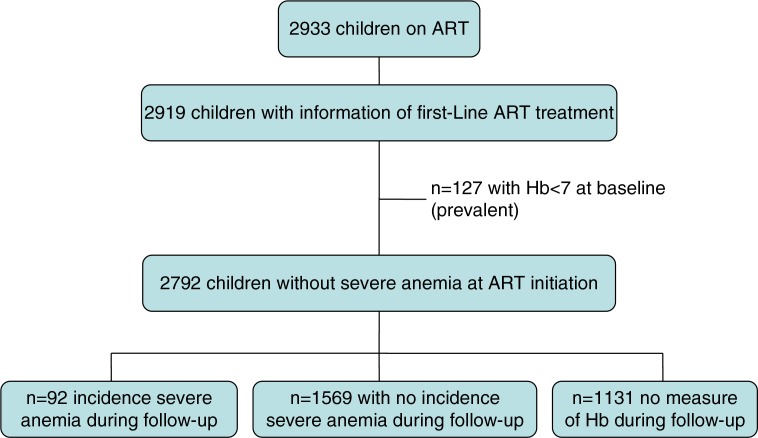
Flow diagram of the paediatric cohort enrolled in the IeDEA Paediatric West Africa Programme.

Of 2933 children on ART, 2919 had information on first-line treatment in the database (Figure 1) and contributed data to this study. A total of 55.1% of the cohort were boys, and 1772 out of 2919 (60.7%) were put on a ZDV-containing regimen.

Baseline Hb concentration was documented in only 2117 (72.5%) children (Table 1). Only four centres use CDC clinical staging hence the very low numbers. The median Hb was significantly higher among children in the ZDV group (median 10 g/dL; IQR: 9–11) compared to children in the non-ZDV group (median 9 g/dL; IQR: 8–10), *p*<0.01 (Table 2). A total of 1281 (60.5%) children had an Hb level <10 g/dL and 127 (6.0%) had severe anaemia, with 69 (54.3%) boys and 58 (45.7%) girls (*p*=0.85). At baseline, 3% of the ZDV group were severely anaemic compared to 10% of the non-ZDV group (*p*<0.01) and these children were excluded from the follow-up analysis. The median CD4 cell count and percentage respectively at baseline were 385 cells/mm^3^ (IQR: 148–695) and 13% (IQR: 7–19); there were no significant differences observed in the immunological profile between the treatment groups. There was no significant difference in median weight-for-age *z*-score between the groups.

**Table 1:** Completeness of data according to first-line treatment

	First-line ART with ZDV (*n*=1772)	First-line ART without ZDV (*n*=1147)	Total (*n*=2919)	*p*
Haemoglobin available *n*, (%)	1244 (70.2)	873 (76.1)	2117 (72.5)	<0.01
CD4 cell count available *n*, (%)	1275 (71.9)	771 (67.2)	2046 (70.1)	<0.01
CD4 cell percentage available *n*, (%)	832 (46.9)	573 (50.0)	1405 (48.1)	0.12
WHO clinical stage available *n*, (%)	646 (36.4)	299 (26.1)	945 (32.4)	<0.01
CDC clinical stage available *n*, (%)	294 (16.6)	189 (16.5)	483 (16.5)	0.96
Clinical staging available *n*, (%)	864 (48.8)	478 (41.7)	1342 (46.0)	<0.01

**Table 2:** Baseline characteristics of children according to the first-line treatment (ZDV-containing ART or not)

	First-line ART with ZDV (*n*=1772)	First-line ART without ZDV (*n*=1147)	Total (*n*=2919)	*p*
Boy *n*, (%)	968 (54.6)	640 (55.8)	1608 (55.1)	0.53
Median age (years) (IQR)	5 (2–8)	6 (2–9)	5 (2–9)	<0.01
First-line initiated *n*, (%)				<0.01
2NRTI +1NNRTI	1262 (71.2)	732 (63.8)	1994 (68.3)	
2NRTI+1IP	465 (26.2)	375 (32.7)	840 (28.8)	
3NRTI	30 (1.7)	5 (0.4)	35 (1.2)	
Median haemoglobin (IQR)	10 (911)	9 (810)	10 (811)	<0.01
Haemoglobin *n*, (%)				<0.01
Hb <7 g/dL *n*, (%)	38 (3.0)	89 (10.2)	127 (6.0)	
Hb [7–10[g/dL *n*, (%)	630 (50.6)	524 (60.0)	1154 (54.5)	
Hb ≥10 g/dL *n*, (%)	577 (46.3)	260 (29.8)	837 (39.5)	
Median CD4 cell count (IQR)	391 (154694)	373 (140710)	385 (148695)	0.99
CD4 cell count *n*, (%)				0.19
<200	374 (29.3)	245 (31.8)	619 (30.2)	
200–500	418 (32.8)	224 (29.0)	642 (31.4)	
≥500	483 (37.9)	302 (39.2)	785 (38.4)	
Median CD4 percentage (IQR)	12 (718)	13 (720)	13 (719)	0.50
CD4 cell percentage *n*, (%)				0.54
<15%	501 (60.2)	330 (57.6)	831 (59.1)	
[15–25%]	227 (27.3)	162 (28.3)	389 (27.7)	
≥25%	104 (12.5)	81 (14.1)	185 (56.6)	
WHO clinical stage IV *n*, (%)	83 (12.8)	31 (10.4)	114 (12.1)	0.28
CDC clinical stage AIDS *n*, (%)	115 (39.1)	97 (51.3)	212 (43.9)	<0.01
Clinical stage *n*, (%)				0.05
Stage I, II, III or stage A, B	681 (78.8)	354 (74.1)	1035 (77.1)	
AIDS or stage WHO IV	183 (21.1)	124 (25.9)	307 (22.9)	
Median weight-for-age *z*-score (IQR)	−2.6 (−4.2–1.4)	−2.7 (−4.4–1.4)	−2.7 (−4.3–1.4)	0.53

Overall, adherence to monitoring guidelines of Hb at baseline was 72.5% complete (Supplemental figure). Follow-up Hb was available for 59.4% of patients. The median duration of follow-up was nine months (IQR 2–23) for the ZDV group and seven months (IQR: 2–25) for the non-ZDV group.

**Figure 2:**
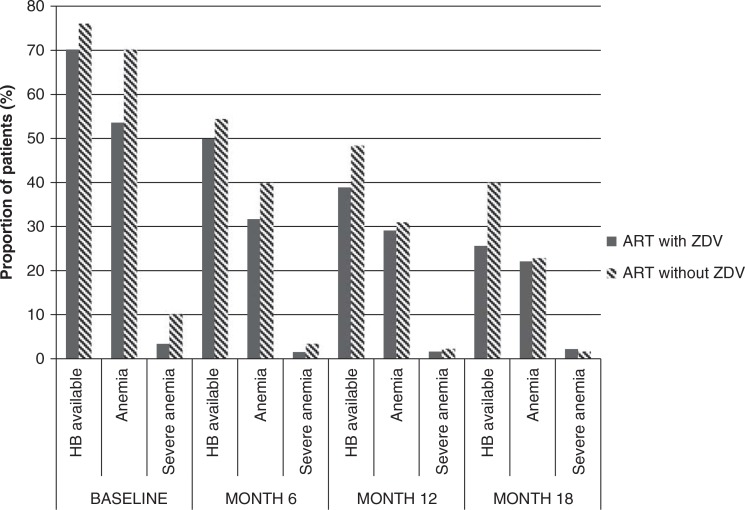
Follow-up data, haemoglobin monitoring and anaemia, according to the first-line treatment (ZDV-containing ART or not).

There were significant differences observed in monitoring during the follow-up period according to ART regimen. At baseline, we observed 70.2% of Hb measurements for the ZDV group versus 76.1% for the non-ZDV group (*p*<0.01). By month 12, we observed 38.9% versus 48.4%, respectively (Figure 2).

Of the 1661 children for whom follow-up Hb was documented, 92 (5.5%) developed severe anaemia, of which 50% were male. By month 6, 3.5% of the non-ZDV group were severely anaemic compared to 1.5% of the ZDV group, *p*≤0.01, and this trend was again observed at 12 months though the difference was not statistically significant, that is, 2.3% versus 1.6%, respectively, *p*=0.41 (Figure 2). There was a statistically significant incidence of severe anaemia of 2.47 [95% CI (2.39–2.54)] and 4.25 [95% CI (4.13–4.37)] per 100 child-years of follow-up for the ZDV and the non-ZDV groups, respectively, *p*<0.01 (Table 3). The incidence of severe anaemia was 2.73 [95% CI (2.65–2.80)] in boys to 3.76 [95% CI (3.65–3.87)] in girls.

**Table 3:** Follow-up according to the first-line treatment (ZDV-containing ART or not)

	First-line ART with ZDV (*n*=1772)	First-line ART without ZDV (*n*=1147)	Total (*n*=2919)	*p*
Median duration of the first-line initiated (months) (IQR)	9 (2–23)	7 (2–25)	8 (2–24)	0.60
Without severe anaemia at baseline (*N*=2792)				
Number at risk of severe anaemia *n*, (%)	1734 (97.8)	1058 (92.2)	2792 (95.6)	<0.01
At least one measure of Hb during follow-up *n*, (%)	1022 (58.9)	639 (60.4)	1661 (59.5)	0.45
Cumulative incidence of severe anaemia during first-line ART (*N*=1661) *n*, (%)	44 (4.3)	48 (7.5)	92 (5.5)	<0.01
Incidence rate of severe anaemia (per 100 child-year of FU) (95% CI)	2.47 (2.39–2.54)	4.25 (4.13–4.37)	3.16 (3.10–3.22)	<0.01
Outcome at the end of the first-line ART initiated *n*, (%)				
Death	88 (5.0)	80 (7.1)	168 (5.8)	0.02
Severe anaemia among deaths	5 (5.7)	7 (8.7)	12 (7.1)	0.65
LTFU	426 (24.0)	167 (14.6)	593 (20.3)	<0.01
Severe anaemia among LTFU	6 (1.4)	1 (0.6)	7 (1.2)	0.05
Active FU	698 (39.4)	333 (29.0)	1031 (35.3)	<0.01
Severe anaemia among active FU	18 (2.6)	22 (6.6)	40 (3.9)	0.63

The loss to follow-up (LTFU) rate of 24% among the ZDV group as compared to 14.6% in the non-ZDV group was statistically significant, *p*<0.01, with a documented incidence of severe anaemia in the LTFU group at last recorded Hb. However, it approached significance (*p*=0.05) at 1.4% (ZDV) and 0.6% (non-ZDV).

**Figure 3:**
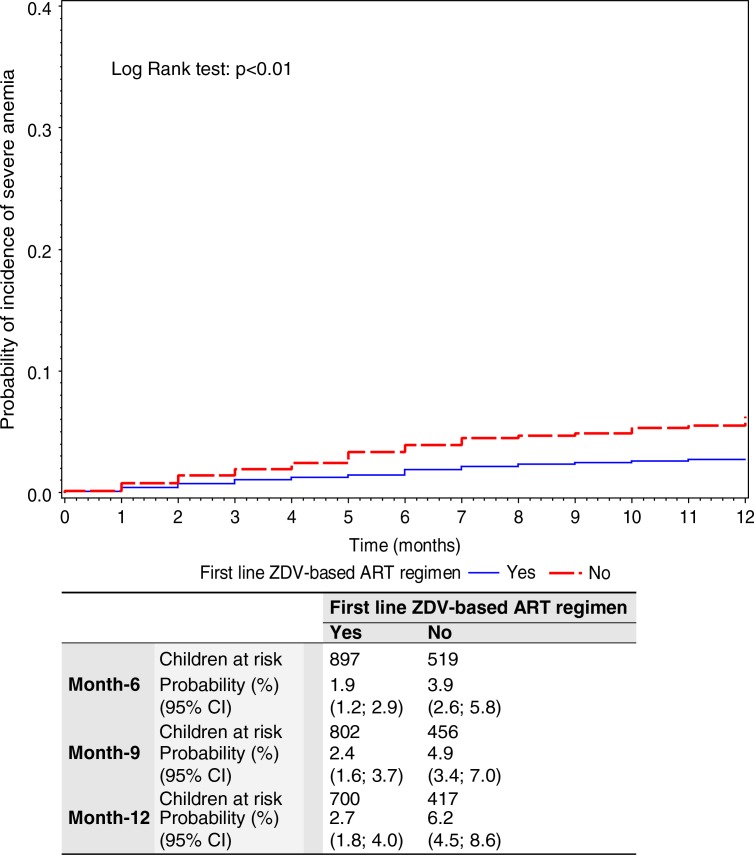
Kaplan–Meier cumulative probability of incidence of severe anaemia during the first-line ART regimen, in 1661 children on ART with one or more measure of haemoglobin during follow-up.

The cumulative probability of severe anaemia increased to 4% over the 12-month follow-up period. On stratifying according to the initial ART regimen, the non-ZDV group had a higher cumulative probability of developing severe anaemia of 6.2% (95% CI: 4.5–8.6) compared to 2.7% (95% CI: 1.8–4.0) in the ZDV group with log-rank *p*≤0.01 (Figure 3).

Assessing the predictors of severe anaemia, children between 12 and 36 months of age had a 1.94 times risk [95% CI (1.20–3.13)] of severe anaemia in the univariate analysis and 2.1 times [95% CI (1.3–3.4)] in the adjusted analysis compared to children above 60 months of age (Supplementary Table).

The baseline clinical stage IV/AIDS, CD4 cell count of <500 and CD4 cell percentage of <15% were not associated with a significant increase in severe anaemia. Children on a non-ZDV-based regime had a significant risk of severe anaemia of 1.85 [95% CI (1.22–2.79), *p*<0.01] on analysis. Adjusted for age at ART initiation and first-line regimen, a weight-for-age *z*-score lower than −3 was a strong predictor [relative risk: 5.59, 95% CI 2.63–11.9; *p*<0.01)] of severe anaemia.

## Discussion

In resource-poor countries such as in West Africa, anaemia is a common disease among children [9–12]. Anaemia in children with HIV may be due to malnutrition, malaria, worm infestation, sickle cell disease, opportunistic infections as well as the HIV infection itself [2, 3, 13]. Prevalence of anaemia of 60.5% at baseline is in keeping with that reported from global studies in children from Western and tropical countries [2, 3, 13–15]. The determinants of anaemia at baseline in this cohort were not identified. The low median weight-for-age *z*-score of −2.7 at baseline was similar in both groups and worse than in the Cross Continents Collaboration for Kids study (3Cs4kids) where the median weight for age was −1.9 SD and median Hb was 10.0 g/dL [16]. In patients who are severely anaemic at baseline, an alternative NRTI to ZDV is recommended by the World Health Organization [4]. In the cohort from this study, the percentage with severe baseline anaemia (6%) was similar to other studies from the same region [14, 17]. Higher prevalence of severe anaemia in the non-ZDV group demonstrates physician compliance with recommended first-line treatment to avoid ZDV. Of note, the children who were severely anaemic at baseline in this cohort were excluded from the follow-up analysis as the study's major objective was to determine progression to severe anaemia.

Although the LTFU rate in the ZDV group was higher, the incidence of severe anaemia was not. The reasons for the LTFU were not documented. In Malawi, LTFU was associated with a mortality rate of 41% [18]. Other reasons include changes in caregivers, difficulties in geographical access to care, drug unpalatability [19, 20] and possible “silent transfers” to other clinics [21] The LTFU rate was high compared to other large multi-centre studies such as the IeDEA Southern Africa Collaboration and the Kids ART LINC collaboration [22, 23] in Africa. Amongst the children who were recorded as dead, a significant percentage was in the non-ZDV group and although there was also a higher percentage of severe anaemia among them, this was not significant. Other studies from Zambia and Kenya have shown that apart from severe anaemia, causes of death amongst African children include malnutrition, pneumonia, cardiac causes, tuberculosis and gastroenteritis [24, 25].

Poor documentation, as in many retrospective observational studies is a limitation of this study. This may have been due to incomplete data entry on the part of the data managers at the centres or clinical shortcomings with physicians not requesting the required laboratory tests. Hb levels are essential in clinical care settings as the presence and severity of anaemia affects choice of ART and clinical management. While technical barriers can explain the absence of baseline CD4 data, the absence of documented baseline clinical staging suggests a more pervasive data collection challenge in this setting. The importance of regular laboratory monitoring in accordance with WHO guidelines [4] needs emphasis in clinical programmes and effective supervision of data entry is essential as anaemia in children is associated with increased morbidity and mortality [2, 22, 24–26]. However, the recently published ARROW study rather emphasized the importance of clinical monitoring with targeted laboratory investigations [5]. An IeDEA paediatric working group global survey reported 90% regular monitoring in West Africa [27]. The pWADA collaboration seeks to improve data quality amongst the participating centres by strengthening data management and disseminating best practices between centres facing similar challenges in this area.

The non-ZDV group had a higher cumulative probability of incidence of severe anaemia during the first year of follow-up. A trend of increasing incidence of severe anaemia in the ZDV group, becoming more apparent by 18 months of follow-up compared to the non-ZDV group was evident. Therefore over time, ZDV may contribute to the incidence of anaemia. Despite this, the cumulative probability of severe anaemia in the cohort overall was however higher in the non-ZDV group. This may be due mainly to the higher proportion being anaemic at baseline in this group. While written care protocols and published guidelines suggest clinicians avoid using ZDV in the presence of anaemia this was not specifically investigated in this study. Other large studies in children and adults including global meta-analyses, evidence from sub-Saharan Africa and India, have reported comparatively worsening levels of Hb on ZDV-containing ART [2, 6, 27–34]. Studies also show that anaemia does not preclude the use of ZDV-containing therapy as the Hb improves over time with immunologic recovery both in children in India and adults in a large cohort in Uganda and the United States [14, 17, 35]. Findings showed that a non-ZDV-containing regimen was significantly associated with severe anaemia when compared to a ZDV-containing regimen. In this cohort, the prevalence of anaemia in general showed a significant reduction in both groups from 53.6% to 5.6% in the ZDV group and 70.2% to 9.2% in the non-ZDV group at baseline and 18 months, respectively. This corroborates the evidence from studies that showed improvement in Hb over time. The risk of severe anaemia is reduced when HIV-positive patients are switched to ZDV after having been stabilized on alternative nucleoside reverse transcriptase inhibitors [28, 31, 33].

Children under five years of age are more vulnerable to severe anaemia as studies from developing countries indicate [14, 36] and the relative risk of severe anaemia in this group was higher.

Although children with Hb less than 10 g/dL at baseline had an increased risk of developing severe anaemia, this was not significant. We found that severe baseline malnutrition was a strong predictor of severe anaemia independent of age and ART regimen in keeping with other studies from Asia and Africa [17, 31–33, 37].

Our study has many limitations. Its retrospective nature, gaps in documentation and site monitoring may have affected data quality. There is a lack of information on whether Hb measurements were driven by guidelines or clinical assessment. Absence of data on the aetiology of anaemia in this cohort is a limitation. Additionally, the high LTFU rate leading to uncertainty about severe anaemia after the children were LTFU could have led to skewed findings. ZDV was not prescribed to children most vulnerable to severe anaemia in conformity with WHO guidelines making the overall comparison not entirely uniform. The real-time nature of this study is a strength as it portrays the situation in everyday clinical practice. Another strength of this study is that it is a large multi-centre, multi-national study conducted over a large but similar geographical area and as such, there are not likely to be significant differences in the non-HIV-related causes of anaemia. This however may limit the generalizability of the findings to other regions.

## Conclusions

This study confirms that the baseline prevalence of anaemia in HIV-infected children in West Africa is very high. Clinicians in West Africa are generally guided by the haemoglobin level in prescribing first-line ART in accordance with recommendations. The prevalence of anaemia improves over time in HIV-infected children on ART. Severe anaemia is not a commonly related toxicity even among children treated with ZDV. Children on ART are nonetheless still at risk of becoming severely anaemic and should be monitored. HIV-infected children under five years of age on ART, especially those less than 36 months of age, are at a higher risk of severe anaemia than their older counterparts. The presence of anaemia at baseline, which may require children to be put on a non-ZDV-containing thymidine regime, is a risk factor for the development of severe anaemia. Severe malnutrition at baseline is a strong predictor for developing severe anaemia and interventions to address this should form an integral component of clinical care. The high LTFU rate is of much concern as this may be an indicator of un-notified deaths. Measures should be instituted in clinical centres to help track all defaulting patients.
